# Editorial: Animal models of allergic diseases: from basic to preclinical research

**DOI:** 10.3389/falgy.2024.1526536

**Published:** 2024-12-16

**Authors:** Friederike Jönsson, Diego Pazos-Castro, Margarida Castell, Pål Johansen

**Affiliations:** ^1^ Institut Pasteur, Université Paris Cité, INSERM UMR1222, Antibodies in Therapy and Pathology, Paris, France; ^2^ CNRS, Paris, France; ^3^ Department of Cancer and Microbiology, Lifesome Therapeutics S.L., Madrid, Spain; ^4^ Physiology Section, Department of Biochemistry and Physiology, Faculty of Pharmacy and Food Science, University of Barcelona, Barcelona, Spain; ^5^ Department of Nutrition, Food Science and Gastronomy, University of Barcelona, Food and Nutrition Torribera Campus, Barcelona, Spain; ^6^ Deptartment of Dermatology, University of Zurich & University Hospital Zurich, Zurich, Switzerland

**Keywords:** allergy, animal models, alpha-gal syndrome, mouse model, asthma, peanut, review articles, original articles

**Editorial on the Research Topic**
Animal models of allergic diseases: from basic to preclinical research

Allergic diseases affect millions worldwide, with latest reports showing an increasing prevalence of asthma, dermatitis (1) and IgE-mediated food allergies. Allergy is currently affecting 10% of the US population (2). Despite advances in the field, there remains an unmet need for definitive treatments and a better understanding of the mechanisms underlying disease development and treatments with symptomatic drugs, new biologics, or allergen immunotherapy (AIT). AIT shows promise as a potential long-term solution but sustaining immune tolerance after treatment cessation (3) and undesirable side effects (4) remain a challenge. In this context, animal models have become essential tools, enabling researchers to investigate the complex mechanisms of allergy and evaluate the efficacy of novel therapies before their clinical application. This Research Topic in the section “Mechanisms in Allergy”, focuses on animal models in allergy research, and highlights recent developments across both foundational and preclinical studies. It showcases diverse approaches that enhance our understanding of allergy mechanisms and therapeutic potential. Here, we discuss the contributions made by four research groups. The published articles—two original research studies and two reviews—each advance knowledge and open avenues for future investigations in allergy.

The original research article by Paolucci et al. introduces a new murine model for studying allergic airway inflammation induced by peanut allergens, a pressing issue given the rising prevalence of peanut allergies and their frequent comorbidity with asthma. Peanut allergies, known for severe reactions, pose unique challenges in creating accurate models due to the disease's specific immunological pathways. The authors use C3H mice sensitized intraperitoneally with peanut allergen, followed by intranasal challenges to induce allergic airway inflammation. The model successfully reproduces key features of human allergic asthma, including structural changes such as bronchial smooth muscle hypertrophy and mucus cell hyperplasia. Additionally, the study confirms peanut allergen-specific immunoglobulin production, as well as Th2 and Th1 cytokine responses, showing how sensitization triggers systemic immune responses. This model stands out for replicating both the humoral and cellular immune responses observed in human allergic asthma, providing a foundation for evaluating peanut allergy treatments in the context of respiratory allergies.

The second original research article by Saunders et al. explores an innovative strategy for AIT based on the use biodegradable nanoparticles to induce immune tolerance in alpha-gal syndrome (AGS), a food allergy triggered by tick bites and characterized by IgE responses to galactose-α-1,3-galactose (αGal). This study investigates the effectiveness of prophylactic and therapeutic nanoparticle treatment in a murine model of AGS, highlighting the immune-modulatory effects of encapsulating αGal glycoprotein in nanoparticles. The study's findings reveal that these nanoparticles effectively reduce Th2 cytokine production, αGal-specific IgE, and hypersensitivity responses in sensitized mice, providing evidence that nanoparticle-based AIT may hold potential for managing AGS. Unlike traditional AITs, these nanoparticles target specific allergenic proteins, offering a degree of specificity and sustained tolerance not typically achieved with conventional treatments. This research represents a significant step forward, introducing a new approach to allergy management that could extend beyond AGS where inducing lasting tolerance is critical.

In their review, Radhouani and Starkl delve into adjuvant-independent airway sensitization models, emphasizing the relevance of physiological (adjuvant-independent) allergen exposure routes to better mimic the onset and progression of allergic asthma. They provide an overview of current models, focusing on the interplay between microbial exposure, infections, and asthma development, which is known to impact disease progression and patient outcomes. This aspect is critical, as real-world allergen exposures often occur alongside microbial encounters and may influence sensitization pathways and allergic responses. Their review underscores the importance of physiological exposure models for understanding how environmental factors, such as infection and microbiota, contribute to allergic airway inflammation. By aligning sensitization models more closely with real-world exposures, researchers can uncover insights that are more likely to translate to human disease, thereby enhancing the clinical relevance of findings. This approach offers a valuable perspective for studying interactions between allergies and environmental influences, an area with profound implications for both prevention and treatment strategies.

In their comprehensive review, Feng et al. compare various animal models for type I hypersensitivity, covering both common allergens and specialized models like shrimp tropomyosin-induced hypersensitivity. They discuss models used for asthma, food allergies, and anaphylactic shock, each designed to address different aspects of IgE-mediated hypersensitivity. The review highlights the importance of careful model selection, emphasizing that specific protocol variables, species, and strains influence the extent to which an animal model replicates human symptoms. A unique contribution of this review is its comparison of allergen types—opposing protein allergens and chemical haptens—and its discussion of the advantages and limitations of various sensitization protocols. By presenting this comparative analysis, Feng et al. provide valuable insights for researchers aiming to select the model best suited to answer a specific research question or investigate a specific type of allergic reaction. This guidance is crucial for refining preclinical studies, as model selection influences the translatability of findings to human clinical settings.

In closing, the articles within this Research Topic collectively underscore the importance of animal models in allergy research, each contributing unique insights into sensitization mechanisms and preclinical evaluation of therapeutic interventions. Both the peanut-allergic asthma model and the innovative nanoparticle AIT for AGS, illustrate the depth and versatility of animal models in advancing our understanding of allergic diseases. Integrating findings from physiological sensitization models and nanoparticle-based therapies with conventional allergy research holds promise to advance insights into how immune tolerance may be safely sustained. Additionally, as environmental and microbial factors continue to shape allergy prevalence and severity, models that explore these variables will be essential to developing prevention strategies and treatments aligned with real-world exposures.

This Research Topic has brought together valuable contributions that expand our knowledge and capabilities in allergy research. By enhancing our understanding of allergen-specific immune responses and developing novel AIT approaches, these studies pave the way toward addressing the unmet clinical needs of allergy patients. The insights gained here highlight that animal models are not only used for discovery but are also key to bridging the gap from bench to bedside, potentially reshaping the future landscape of allergy treatment and prevention.
